# Hepatic encephalopathy in ICU: cerebrospinal fluid metabolomics highlights alteration of multiple metabolic pathways representing new potential therapeutic targets

**DOI:** 10.1186/2197-425X-3-S1-A690

**Published:** 2015-10-01

**Authors:** N Weiss, B Colsch, F Isnard, S Attala, MDM Amador, F Lamari, F Sedel, C Junot, D Thabut, Brain Liver Pitie-Salpetriere Study Group (BLIPS)

**Affiliations:** Assistance Publique - Hopitaux de Paris, La Pitié-Salpetriere Hospital, Neurological ICU, Neurology Department, France, France; CEA, Saclay, France; Assistance Publique - Hopitaux de Paris, La Pitié-Salpetriere Hospital, Neurology Department, La Pitié-Salpêtrière Hospital, Paris, France; Assistance Publique - Hopitaux de Paris, La Pitié-Salpetriere Hospital, Paris France; CEA, Scalay, France; Assistance Publique - Hopitaux de Paris, La Pitié-Salpetriere Hospital, Hepatological ICU, Paris, France

## Introduction

Hepatic encephalopathy (HE) is a neurological complication of cirrhosis, impairing survival and quality of life. Its incidence is growing because of the improved prognosis of other complications of cirrhosis, and of the widespread use of TIPS. However, besides hyperammonemia which is often pointed out as a cause of HE, the pathophysiological mechanisms of HE remains poorly understood, which prevents the development of therapeutic strategies. To address this issue, metabolomics was used to identify dysfunction of metabolic pathways in cerebrospinal fluid (CSF) samples of cirrhotic patients suffering from HE.

## Objectives

The aim of this study was to detect new therapeutic targets for HE associated with cirrhosis.

## Methods

Cerebrospinal Fluid (CSF) samples were collected on 14 cirrhotic patients admitted in ICU for HE, in whom infection of central nervous system has to be ruled out, and were compared to CSF of 27 control patients without any proven neurological disease. Metabolomic analysis was performed using 3 liquid chromatographies coupled to high resolution mass spectrometry methods (LC-HRMS). Informatic data processing tools were used.

## Results

LC-HRMS methods led to the characterization of 150 metabolites in CSF samples of HE patients, which were mainly amino acids and organic acids. Interestingly, according to human metabolome database, 40% of those metabolites had never been retrieved were not reported as present in CSF before. HE patients could be easily discriminated from controls on the basis of metabolomicic information. Concentrations of 102 metabolites were found to be significantly altered in HE patients: metabolotypes displayed alterations in several major metabolite classes such as ammoniac, bile acids, but also amino-acids, acylcarnitines, and nucleosides. Accumulations of acetylated compounds, which could be due to a defect of the Krebs cycle, were reported for the first time in HE patients, and could constitute interesting therapeutic targets.

## Conclusions

By enabling the simultaneous monitoring of a large set of metabolites in cirrhotic patients with HE, CSF metabolomics highlighted several altered metabolite pathways linked to ammonia metabolism, neurotransmission and energy metabolism. The pharmacological relevance of our findings has to be explored on animal models, as they could constitute interesting new therapeutic targets.

